# Nuclear receptor retinoid-related orphan receptor alpha promotes apoptosis but is reduced in human gastric cancer

**DOI:** 10.18632/oncotarget.14364

**Published:** 2016-12-29

**Authors:** Zhengguang Wang, Fangyuan Xiong, Xiaoshan Wang, Yijun Qi, Haoyuan Yu, Yong Zhu, Huaqing Zhu

**Affiliations:** ^1^ Department of Surgery, First Affiliated Hospital of Anhui Medical University, Hefei, Anhui, P.R. China; ^2^ Laboratory of Molecular Biology and Department of Biochemistry, Anhui Medical University, Hefei, Anhui, P.R. China

**Keywords:** gastric carcinoma, RORα, AMPK, apoptosis, chemotherapy resistance

## Abstract

Retinoid-related orphan receptor α (RORα) is a nuclear receptor, which regulates inflammation and immune responses, lipid metabolism and circadian rhythm. Although RORα suppresses breast tumor invasion, it is unknown whether RORα is dysregulated in gastric cancer leading to cellular survival. Therefore, we hypothesize that RORα is dysfunctional in gastric carcinoma and this causes decreased apoptosis in gastric cancer cells. To test this hypothesis, we employed human gastric cancer tissues with different stages to determine RORα expression, as well as *in vitro* human gastric cancer cells to determine how RORα is reduced during apoptosis. We found that the expression of RORα was reduced in gastric tissues with cancer, and this correlated with increased TNM stages. The mechanisms underlying RORα reduction is due to the reduced activation of AMP-activated protein kinase (AMPK), as a selective AMPK activator AICAR increased RORα activation and level in human gastric cancer cells. Furthermore, AICAR treatment increased RORα recruitment on the promoters of tumor suppressor genes (i.e., FBXM7, SEMA3F and p21) leading to apoptosis in human gastric cancer cells. Taken together, RORα reduction occurs in gastric cancer leading to the survival of tumor cells, which is attenuated by AMPK. Therefore, both RORα and AMPK are potential targets for the intervention and therapy in gastric carcinoma.

## INTRODUCTION

Gastric cancer is the fourth most common cancer and is the third leading cause of cancer, with more than 700,000 deaths every year all over the world [1–3]. Despite the declined incidence and mortality due to the major improvements in diagnosis and treatment, there are less than 20% of patients with gastric cancer surviving up to 5 years [1]. Gastric cancer is usually treated with chemotherapy and surgery, but chemoresistance seriously hinders the treatment of gastric cancer. Therefore, it is an urgent to develop a novel chemotherapy or chemosensitizer in enhancing the chemosensitization.

Retinoic acid-related orphan receptor alpha (RORα) encoded by NR1F1 gene is a nuclear receptor in the ROR sub-family [4]. It is well-known that RORα regulates inflammation and immune responses, lipid metabolism and circadian rhythm [5–8]. Recent studies have shown that RORα is associated with cancer prognosis through the modulation of cell proliferation [9–13]. However, there are no reports regarding the regulation of RORα in gastric cancer and whether RORα modulates apoptosis in gastric cancer cells. We hypothesize that RORα is dysregulated in gastric cancer and this dysregulation reduces the apoptosis in gastric cancer cells. To test this hypothesis, the mRNA and protein levels were determined in human gastric cancer tissues with different stages. We also employed the gastric cancer cell lines to determine the mechanisms for RORα dysregulation and whether RORα promotes apoptosis.

## RESULTS

### Expression of RORα was reduced in human gastric cancer tissues

**Figure 1:**
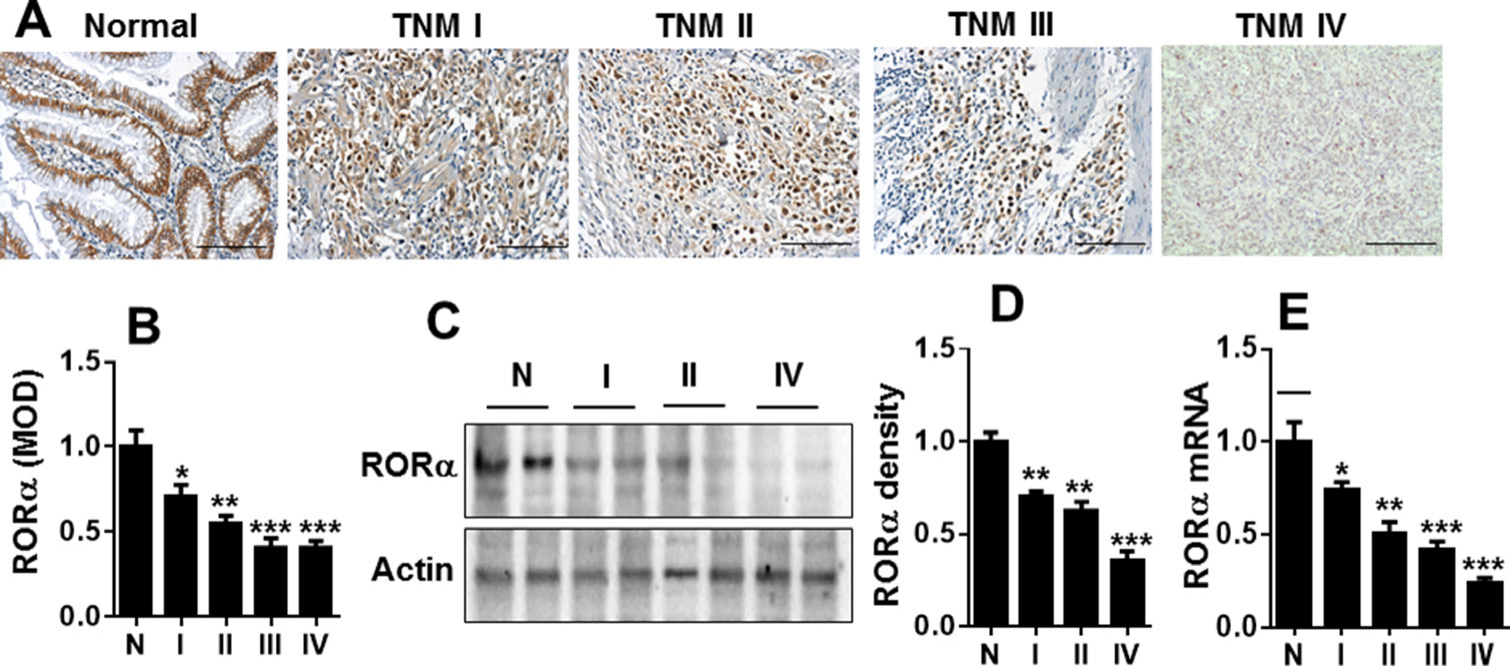
RORα was reduced in human gastric cancer (**A**) Representative IHC results showing the expression of RORα in human gastric tissues adjacent to tumor (Normal, N) and with cancer from TNM I to TNM IV. Bar size: 100 μM. (**B**) The MOD showing the changes in RORα expression by immunohistochemistry in A. (**C**) Representative Western blots showing the changes of RORα levels in human gastric tissues adjacent to tumor (Normal, N) and with cancer from TNM I to TNM IV. β-actin was used a loading control. (**D**) The densitometry of RORα bands in C. Relative protein expression of RORα was normalized to that of β-actin. (**E**) The levels of RORα mRNA in human gastric tissues adjacent to tumor (Normal, N) and with cancer from TNM I to TNM IV, which was measured by qPCR. 18S rRNA was used a housekeeping gene. Data are expressed as the mean ± SEM. *N* = 3–6. *P < 0.05, **P < 0.01, ***P < 0.001, vs. Normal.

To test the role of RORα in human gastric cancer, the abundance of RORα in gastric tissues adjacent to cancer (normal) and with different clinical stages of gastric carcinoma was determined by immunohistochemistry. As shown on Figure 1A and 1B, the expression of RORα was significantly reduced in gastric cancer tissues compared to non-cancer gastric tissues. The reduction of RORα abundance was associated with disease stage. Similarly, the reduction of RORα abundance in gastric cancer tissues was confirmed by Western blot (Figure 1C and 1D). Furthermore, we measured RORα mRNA level in gastric cancer tissues and matched adjacent gastric mucosa. In consistent with the above findings, the expression of RORα mRNA was significantly down-regulated in gastric cancer compared with matched adjacent gastric mucosa (Figure 1E). These results suggest the significant reduction of RORα in gastric cancer tissues, which is associated with the clinic stage.

### Association of RORα expression with clinicopathological factors

To determine the clinical significance of RORα, we analyzed the correlations between the RORα level and clinicopathological factors in according to immunohistochemistry results (Table 1). Low expression of RORα protein was significantly associated with tumor size, tumor differentiation, T stage, TNM stage, and lymph node metastasis. The results indicate that RORα level is associated with the progression and prognosis of gastric cancer.

**Table 1:** Relationship of RORα expression to clinicopathological variables

Variables		Numbers of patients (*n* = 74)	Low RORα levels (*n* = 43)	High RORα levels (*n* = 31)	*P* value
Sex	Male	58	35	23	0.458
	Female	16	8	8	
Age (years)	< 60	23	16	7	0.180
	≥ 60	51	27	24	
Primary tumor site	Gastric cardia	33	20	13	0.489
	Gastric antrum	21	9	12	
	Gastric body	17	12	5	
	Gastric fundus	3	2	1	
Diameter of tumor	< 5 cm	31	13	18	0.017
	≥ 5 cm	43	30	13	
Adenocarcinoma	Moderately differentiated	22	8	14	0.014
	Poorly differentiated	52	35	17	
T stage	T1	9	0	9	0.001
	T2	6	3	3	
	T3	38	24	14	
	T4	21	16	5	
TNM stage	I	11	3	8	0.023
	II	7	2	5	
	III	32	23	9	
	IV	24	15	9	
Lymph node metastasis	Present	58	38	20	0.009
	Absent	16	5	11	

### Expression of RORα was reduced in human gastric cancer cell lines

**Figure 2:**
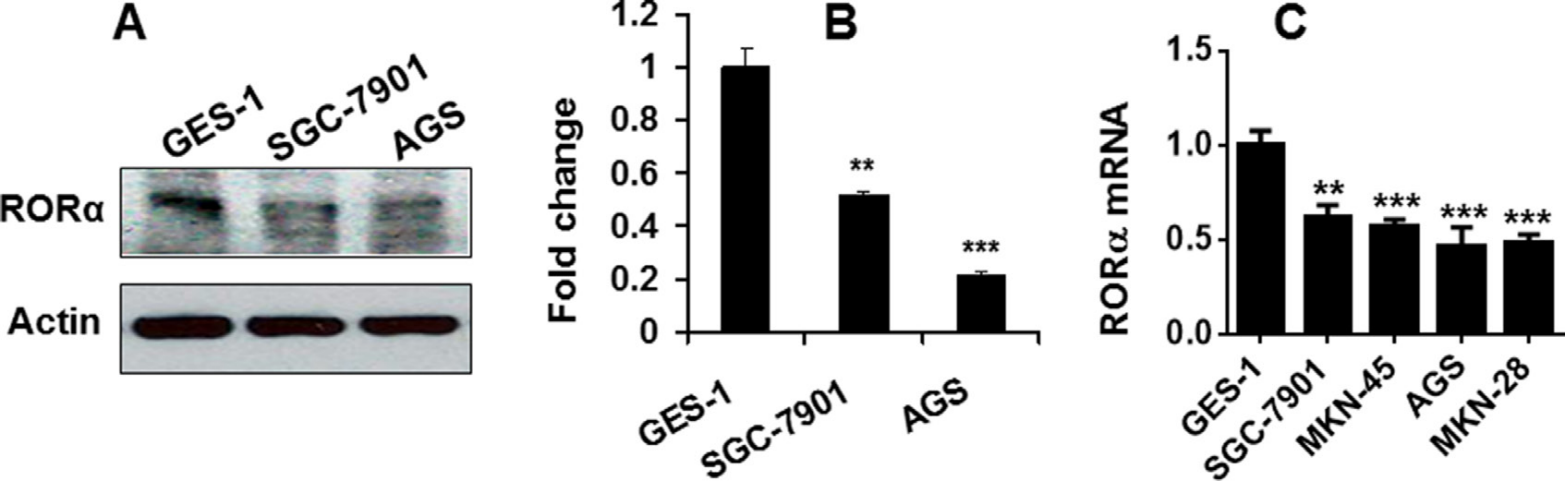
RORα was reduced in human gastric cancer cell lines (**A**) RORα protein levels determined by Western bolts in human gastric epithelial cells (GES-1) and gastric cancer cell lines (SGC-7901 and AGS). β-actin was used a loading control. (**B**) The densitometry of RORα bands in A. Relative protein expression of RORα was normalized to that of β-actin. (**C**) RORα mRNA expression determined by qPCR in human gastric epithelial cells (GES-1) and gastric cancer cell lines (SGC-7901, MKN-45, AGS, and MKN-28). 18S rRNA was used a housekeeping gene. Data are expressed as the mean ± SEM. *N* = 3–6. **P < 0.01, ***P < 0.001, vs. GES-1 cells.

To further determine the association of RORα with gastric cancer, we employed human gastric cancer cell lines. As shown in Figure 2A and 2B, the protein levels of RORα determined by Western blot were significantly reduced in human gastric cancer cells SGC-7901 and AGS cells as compared to normal gastric epithelial cells GES-1. Similarly, the mRNA level of RORα was decreased in SGC-7901, AGS, MKN-28 and MKN-45 compared with GES-1 cells (Figure 2C). These data further demonstrate the significant reduction of RORα in gastric cancer cells.

### AMP-activated protein kinase (AMPK) promoted RORα activation and levels in gastric carcinoma cells

**Figure 3:**
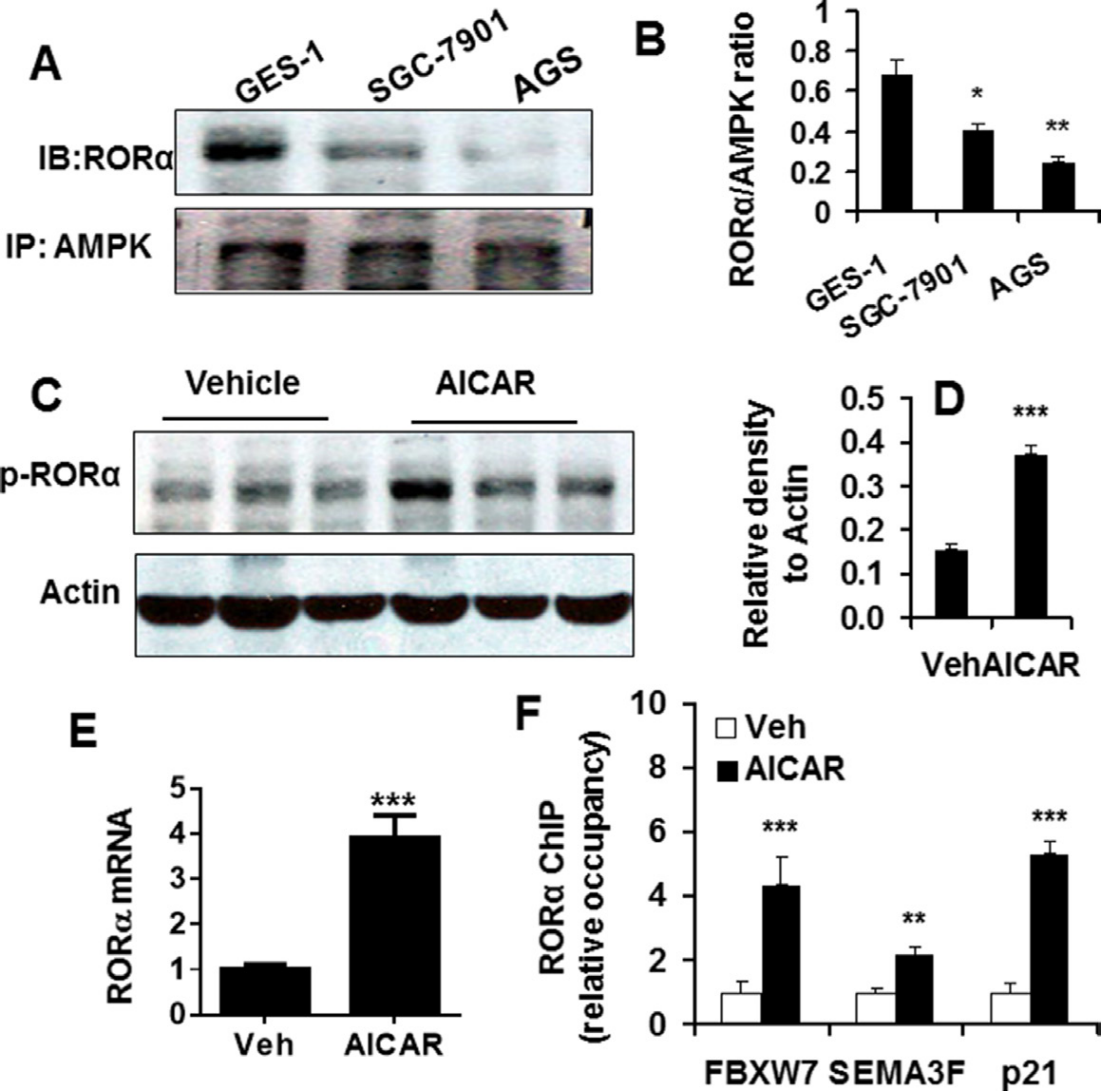
AMPK interacted RORα and regulated its activity and levels (**A**) Physical interaction of RORα (IB) with AMPK (IP) was determined by co-immunoprecipitation, and representative bands were shown. (**B**) Densitometry of RORα to AMPK ratio was shown. Data are expressed as the mean ± SEM. *N* = 3. *P < 0.05, **P < 0.01, vs. GES-1 cells. (**C**) RORα phosphorylation determined by Western blot in SGC-7901 cells treated with AICAR (1 mM for 48 h). β-actin was used a loading control. (**D**) The densitometry of RORα phosphorylation bands after normalization with β-actin. ***P < 0.001, vs. vehicle. (**E**) RORα mRNA determined by qPCR in SGC-7901 cells treated with AICAR (1 mM for 48 h). 18S rRNA was used a housekeeping gene. ***P < 0.001, vs. vehicle (Veh). (**F**) Changes in RORα recruitment on the promoters of FBXW7, SEMA3F, and p21 genes, which is determined by ChIP, in SGC-7901 cells treated with AICAR (1 mM for 48 h). ChIP analysis was performed using RORα antibody or normal serum IgG (as control) as described in materials and methods. **P < 0.01, ***P < 0.001, vs. corresponding vehicle (Veh) controls.

We and others have shown that AMPK reduction in gastric cancer, which regulates cancer cell proliferation and apoptosis [14–16]. Hence, we hypothesized that AMPK modulates RORα activity and level. To test this hypothesis, we first employed the co-immunoprecipitation (Co-IP) approach to detect the physical interaction of AMPK and RORα in SGC-7901 cells. As shown in Figure 3A and 3B, the interaction of AMPK and RORα was observed in normal gastric epithelial cells GES-1. This ratio of RORα to AMPK blot was significantly reduced in SGC-7901 and AGS cells. Next we treated SGC-7901 cells with a selective AMPK activator AICAR (1 mM, 48 h), and then determined RORα phosphorylation by Western blot. As shown in Figure 3C and 3D, AICAR treatment significantly increased the phosphorylation of RORα (Ser 35), suggesting its activation [11]. Furthermore, we also observed increased levels of RORα mRNA in SGC-7901 cells treated with AICAR (1 mM, 48 h) (Figure 3E).

AMPK has been shown to increase the expression of tumor suppressor genes including F-box and WD repeat domain containing 7 (FBXW7), semaphorin III/F (SEMA3F), and p21^Cip1^ (p21) in gastric cancer cells, and RORα functions a transcription activator [14, 17]. Therefore, we hypothesized that RORα activation by AMPK enhances its recruitment on the promoters of these genes. The chromatin immunoprecipitation (ChIP) was performed to determine the recruitment of RORα on the promoters of FBXW7, SEMA3F, and p21 genes in SGC-7901 cells treated with AICAR (1 mM, 48 h). As shown in Figure 3F, AICAR treatment enhances the recruitment of RORα on the promoters of FBXW7, SEMA3F, and p21 genes in SGC-7901 cells. Altogether, these results suggest that the RORα reduction in gastric cancer is possible due to the decrease in AMPK, which leads to its recruitment on tumor suppressor genes.

### Effect of RORα on the apoptosis in SGC-7901 cells

**Figure 4:**
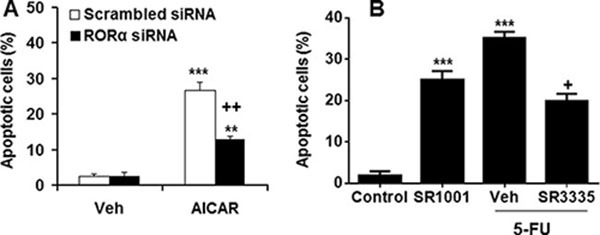
RORα regulated apoptosis in human gastric cancer cells (**A**) Transfection with RORα siRNA reduced apoptosis in SGC-7901 cells induced by AMPK activator AICAR (1 mM for 48 h). **P < 0.01 and ***P < 0.001 vs. vehicle (Veh); ^++^*P* < 0.01 versus Scrambled siRNA/AICAR group. (**B**) Treatment of RORα agonist (SR1001) induced apoptosis, whereas RORα reverse agonist (SR3335) attenuated 5-FU-induced apoptosis in SGC-7901 cells. ***P < 0.001 vs. control; ^+^*P* < 0.05 versus 5-FU alone.

To determine the role of RORα in apoptosis, we transfected its siRNA into SGC-7901 and measured the apoptosis using a photometric ELISA assay. As shown in Figure 4A, transfection with RORα siRNA significantly decreased AICAR-induced apoptosis in SGC-7901 cells as compared to scrambled siRNA control. Treatment with RORα agonist SR1001 increased apoptosis in SGC-7901 cells, whereas its reverse agonist SR3335 reduced 5-FU-mediated apoptosis (Figure 4B). These data implicate that RORα promotes apoptosis in human gastric cancer cells.

## DISCUSSION

**Figure 5:**
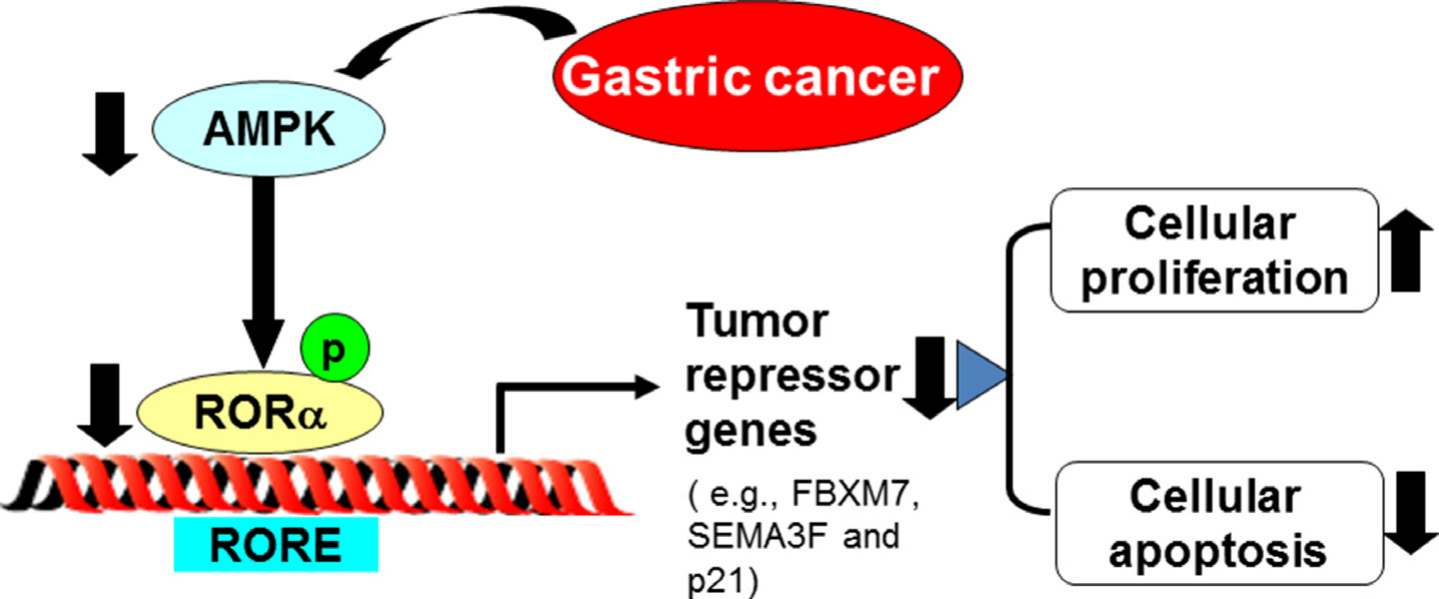
Schematic figure of our findings RORα was down-regulated in human gastric cancer tissues, which was associated with the progression and poor prognosis. This is due to the decreased AMPK, leading to less expression of tumor repressor gene and apoptosis.

In the present study, we have shown the novel role of RORα in human gastric cancer. RORα was down-regulated in human gastric cancer tissues, and this reduction was associated with the progression and poor prognosis. The mechanisms underlying RORα reduction in human gastric cancer were due to the decreased AMPK, leading to less apoptosis (Figure 5).

There are three ROR families including RORα, RORβ, and RORγ, and all are transcriptional activators recognizing ROR-response elements [6]. RORα is expressed in a variety of cell types including gastric epithelial cells [18]. It has been shown that RORα regulates several cellular processes involved in development, circadian rhythm, and inflammatory responses [5–7]. Recent studies suggest the potential role of RORα in progression and prognosis of cancer including colon cancer and breast cancer [11, 19, 20], and that RORα functions as suppression of tumor cell proliferation and augmentation of apoptosis [12, 18, 21–24]. Our study here for the first time demonstrated that RORα was significantly reduced in human gastric cancer tissues, which is associated with the clinical stages and lymph node metastasis. These findings provide the possibility of RORα as a biomarker of severity and prognosis of gastric cancer, although the follow-up experiments to monitor the survival duration of patients with low and high expression of RORα.

RORα plays an important role in increasing apoptosis, which forms a basis for its tumor-suppressive regulatory role [21, 24]. We employed both genetic and pharmacological approaches to determine the role of RORα in regulating apoptosis in gastric cancer cells. We found that RORα activation induced apoptosis, whereas its knockdown by siRNA or pharmacological inhibition reduced apoptosis in gastric cancer cells. This may serve a mechanism for progression of gastric cancer through reduced apoptosis in cancer cells when RORα is significantly ameliorated. Furthermore, our study showed that RORα can be recruited on the promoter of tumor suppressor genes including FBXW7, SEMA3F, and p21. As a transcription activator, RORα reduction may reduce the transcription of these tumor suppressor genes, resulting in cancer progression. This is consistent with the finding showing SEMA3F, a direct RORα target gene with a ROR element, suppresses breast tumor invasion [12, 23]. The ongoing experiment is to investigate whether RORα stabilize p53 therefore modulating apoptosis [24]. Further study is required to determine the role of RORα in regulating proliferation, migration, invasion of gastric cancer cells, and chemotherapy sensitivity, as well as in animal model of gastric cancer. Recent studies have shown that in hepatoma cells RORα reprograms glycolysis that is upregulated in gastric cancer for cell proliferation (Warburg effect) [25, 26]. It remains unknown whether RORα reduces glycolysis in gastric cancer thereby reducing their proliferation.

In the present study, we found that RORα can be posttranslationally regulated by AMPK, as AMPK activator AICAR treatment increased RORα phosphorylation (Ser35). This is further confirmed by the experiment showing the physical interaction of RORα with AMPK in normal gastric epithelial cells. This interaction was reduced in gastric cancer cells. The levels of AMPKα are decreased in the early stage of gastric cancer, and patients with gastric carcinoma often have a favorable prognosis with positive expression of AMPK [15, 16, 27, 28], suggesting the reduced RORα phosphorylation/activation in gastric cancer. In addition to phosphorylation, RORα mRNA was also augmented in gastric cancer cells treated with AICAR, although AMPK siRNA or overexpression approaches would further support these data. The mechanisms underlying these findings need to be identified. RORα has been shown to activate AMPK in liver tissues [29], which raises the question whether there have a positive feedback between AMPK and RORα in regulating the progression of human gastric cancer. Overall, these finding suggest that AMPK regulates RORα through both transcription and posttranslational modifications, and that RORα may be a potential novel therapeutic target for AMPK-induced apoptosis in gastric cancer cells.

In summary, RORα is down-regulated in human gastric cancer, which causes the resistance to apoptosis in gastric cancer cells. Mechanistically, AMPK reduction leads to the decreased activation and transcription of RORα, resulting in the expression of tumor suppresser genes. Utilization of RORα agonist or AMPK activator would be a potential therapeutic strategy for the treatment of gastric cancer.

## MATERIALS AND METHODS

### Patients and tissue collection

Eligible 74 patients were adults (18 years old to 75 years old) with biopsy-confirmed gastric adenocarcinoma with histological examination. All enrolled patients underwent total or subtotal gastrectomy since 2014. None of the patients had received radiotherapy or chemotherapy before surgery. After surgery, each patient received chemotherapy with the regimens of FOLFOX4 program. The clinical characteristics were shown in Table 1. All patients had normal hepatic, renal and bone marrow function, as well as ECOG performance status between 0–2. Patients were excluded for serious disorders, peripheral neuropathy (NCI-CTC1 level and above), pregnancy, or breast-feeding.

All patients were screened and treated for the purpose of the study at the Affiliated Hospital of Anhui Medical University, Hefei, China, and signed an informed consent form. The human ethics guidelines was discussed and approved by the Human Ethics Committee in the First Affiliated Hospital of Anhui Medical University [30].

### Immunohistochemistry

The expression of RORα in normal gastric tissues and gastric carcinoma tissues with different clinical stages were measured by immuohistochemistery [30]. The specimens were blocked with 3% hydrogen peroxide, 10% normal goat serum, and then incubated with the RORα antibody (1:500 dilutions, Abcam, USA) overnight at 4^°^C. After treated with biotin-conjugated secondary antibody, the slides were incubated with streptavidin-biotin horseradish peroxidase complex, followed by incubation with diaminobenzidine (DAB, ZSGB-BIO, China) for 5 min. The counterstaining with hematoxylin was then performed, and slides were observed under a bright-field microscope in a double-blinded manner. The integral optical density value of all images was analyzed, and relative protein expression levels were densitometrically calculated and expressed in the mean optical density (MOD) units [30, 31]. The staining intensity was scored as ‘0’ (no staining), ‘1’ (≤ 25%, weakly stained), ‘2’ (25%–50%, moderately stained), or ‘3’ (≥ 50%, strongly stained). A low RORα expression was defined as score ‘0’ or ‘1, and a high RORα expression was defined as score ‘2’ or ‘3’. The patients were then divided into two groups: RORα high expression group (*n* = 31) and RORα low expression group (*n* = 43) (Table 1).

### Cell treatment and transfection

Gastric carcinoma cells (SGC-7901, AGS, MKN-28, and MKN-45) and human gastric epithelial cell line (GES-1) were purchased from the American Type Culture Collection (ATCC, USA). These cells were maintained on tissue culture flask, propagated in DMEM medium with 10% FBS, penicillin (100 U/ml), and streptomycin (100 U/ml) in 5% CO_2_ and humid air at 37^°^C. Cells were split every 2–3 days by trypsinization and centrifugation, followed by aspiration of the culture medium. In a separate experiment, SGC-7901 cells were plated in 6-well plates at a density of 1 × 10^6^ cells/2 ml media, and then treated with a selective activator of AMPK, which was 5-aminoimidazole-4-carboxyamide ribonucleoside (AICAR) (1 mM, Sigma, USA), or with specific RORα agonist (SR1001, 0.5 μM, Cayman, USA) and reverse agonist (SR3335, 0.5 μM, Cayman, USA) for 48 h [14]. To reduce endogenous ROR expression, SGC-7901 cells were seeded onto a 12-well plate (5 × 10^5^/well) and transfected with human RORα siRNA (Dharmacon RNA Technologies, Lafayette, CO, USA) at 50 nM using the Lipofectamine reagent (Invitrogen, Carlsbad, CA, USA) according to the instructions. Twenty-four hours after transfection, cells were treated with vehicle or AICAR (1 mM) for 48 h.

### Western blot analysis

Gastric tissues and cells were lysed in RIPA buffer (Tris-HCl, pH 7.14, 150 mmol/L NaCl, 1 mmol/L EDTA, 1% Triton, 0.1% SDS, 5 mg/ml Leupeptin, and 1 mmol/L PMSF). After repetitive freeze-thawing for 3 times, the lysates were centrifuged at 14,000 rpm for 10 min at 4^°^C. The protein concentration of the sample was measured with Micro-BCA Protein Assay Reagent Kit (Beyotime, China). Protein extracts were separated through 12% SDS–PAGE and transferred to polyvinylidene fluoride membranes. The membrane was blocked with 5% fat-free milk in TBST (PBS with 0.1% Tween 20) for 2 h at room temperature. After washing 3 times (10 min each time) with TBST, the corresponding antibodies against RORα (1:1000 dilutions), p-RORα (1:1000 dilutions), and actin (1:1000 dilutions, Santa Cruz Technology, USA) were incubated overnight at 4^°^C. The membrane was incubated with the appropriate HRP-conjugated secondary antibodies (1:5,000 dilutions, Millipore) for 2 h at room temperature, and detected with enhanced chemiluminescence (ECL, Beyotime, China). Equal loading of the samples was determined by quantification of proteins as well as by reprobing membranes for a housekeeping control β-actin. The ImageJ software was used to quantify the densitometry of Western blot bands.

### Co-IP assay

Gastric epithelial and cancer cells including GES-1, SGC-7901 and AGS lysed with RIPA buffer, and cell lysates were used for AMPK immunoprecipitation with a polyclonal antibody against AMPK (1:40 dilutions, Santa Cruz Biotechnology, USA), which was added to 150 μg of sample proteins in a final volume of 200 μl, and incubated for 1 h. Protein-A/G agarose beads (10 μl) were added to each sample and kept overnight at 4°C on a rotating rocker. For immunoblot, the immunoprecipitated AMPK agarose bead suspension was resolved by SDS-PAGE gradient gels. The membranes were blotted using the RORα antibody. The densitometry of RORα bands was normalized to AMPK with quantitative analysis using the ImageJ software.

### ChIP assay

ChIP was performed according to the protocol as described previously [32, 33]. Briefly, the lysates from SGC-7901 cells treated with an AMPK activator AICAR (1 mM, 48 h) or vehicle were cross-linked with 1% formaldehyde for 10 min. Cell pellets were resuspended with SDS-lysis buffer containing 50 mM Tris-HCl, 1% SDS, 5 mM EDTA, 5 mM sodium butyrate, and protease inhibitors, and sonicated four times for 30 sec at a maximum speed using a Sonicator. Supernatants were precleared t with 60 μl of protein A agarose/salmon sperm DNA for 3 h at 4°C [33]. After immunoprecipitation with RORα antibody (1 μg) overnight, 40 μl of protein A agarose/salmon sperm DNA was added and incubated for 2 h. Precipitates were washed sequentially with Paro buffer I, Paro buffer II, and Paro buffer III for 5 min at 4°C. The antigen-antibody complexes were extracted with 50 μl elution buffer (0.2 μg/μl proteinase K, 1% SDS, and 0.1 M NaHCO_3_). The eluted samples were incubated at 65°C overnight to reverse formaldehyde cross-linking. The recovered DNA was purified with a QIAquick PCR purification kit (Qiagen, Valencia, CA, USA). Samples of input DNA were also prepared in the same way as described above. Real-time PCR was performed to determine the expression of tumor suppressor genes FBXW7, SEMA3F, and p21 as described below.

### Quantitative real-time PCR

Total RNA was isolated using TRIzol (Invitrogen, USA) according to the manufacturer's instructions. The cDNA was generated using a Transcriptor first-strand cDNA synthesis kit (TaKaRa, Shiga, Japan), and the primers for FBXW7, SEMA3F, and p21 were used for PCR amplification [14, 34–36]. Relative levels of specific mRNA were determined using the Thermo PIKOREAL 96 real-time PCR detection system with QIAGEN SYBR ^®^Green supermix (Valencia, CA, USA) according to the manufacturer's instructions. The 18 rRNA gene was used as an internal control for normalization.

### Apoptosis assay

The percentage of apoptotic cells was determined using a photometric ELISA assay from Boehringer-Mannheim that measures cytoplasmic histone-associated DNA fragments as previous work [37]. Optimal apoptotic response (assuming 100% apoptotic cells) was observed after cell treatment with 50 μM camptothecin for 24 h, and this value was used to calculate the percentage of apoptotic SGC-7901 cells after the treatments.

### Statistical analysis

Statistical analyses were employed by SPSS 19.0. The results were presented as mean ± SEM. One-way analysis of variance (ANOVA) was used for the statistical significance of the differences between groups. The chi-square test was used to analyze the RORα expression to clinicopathological variables. There existed statistical significance when *P* < 0.05.
